# Fractal analysis of hepatocellular carcinoma vasculature shows regional differences independent of vascular invasion

**DOI:** 10.1038/s41598-026-38580-x

**Published:** 2026-02-25

**Authors:** Jake Penney, Victor Nardon, Aurélie Beaufrere, Miguel Albuquerque, Valérie Paradis, Ralph Sinkus

**Affiliations:** 1 Université Paris Cité, Centre de Recherche sur l’Inflammation (CRI), INSERM, U1149, CNRS, ERL 8252, 75018 Paris, France; 2 Siemens Healthcare SAS, 92400 Courbevoie, France; 3 Service de Pathologie, Hôpital Beaujon, FHU MOSAIC, Siric InsiTu, Assistance Publique-Hôpitaux de Paris, 92110 Clichy, France; 4 School of Biomedical Engineering and Imaging Sciences, King’s College London, Newcomen St, London, SE1 1UL UK

**Keywords:** Image processing, Cancer imaging, Imaging techniques, Tumour angiogenesis

## Abstract

Tumor vasculature architecture influences treatment response, particularly in hepatocellular carcinoma (HCC) where anti-angiogenic therapies are standard. Although CD31 is a positive endothelial stain, classical CD31-derived metrics –such as vessel density or area fraction–primarily quantify vascular abundance and provide limited insight into organization. These metrics cannot reliably separate vascular phenotypes across tissue regions when organization, rather than quantity, is the discriminating feature. To address this gap, we investigated whether mathematical, scale-dependent descriptors could distinguish vascular organization beyond what classical metrics capture. Here, fractal dimension and Hurst index were derived on vessels segmented from 29 CD31-stained HCC samples across tumoral, peri-tumoral, and distant non-tumoral regions, with/without vascular invasion–a key prognostic indicator. Statistically significant differences $(p < 0.01)$ in fractal dimension were observed between the three regions between $16-128\mu m$ box size, whereas extremal scales showed no differences. The Hurst index exhibited similar differences emerging within the $20-200\mu m$ range. This fractal disparity reflects underlying differences in vessel-shape distributions, with tumoral regions containing fewer small, round vessels than non-tumorous tissue. Conversely, vascular invasion did not yield significant differences $(p > 0.1)$. These findings demonstrate robust regional differences in vascular organization in HCCs, supporting future studies investigating vascular fractality through non-invasive imaging approaches.

## Introduction

Cancer in general is characterized by an anarchic and chaotic vasculature arising from the haste at which vessels are newly formed to satisfy the metabolic demand of the tumor^1^. Quantifying the spatial organization of the tumor’s vasculature is carrying great potential to gauge the likelihood of response to therapy, in particular for anti-angiogenic or novel agents normalizing vascular architecture^2^, and to potentially even predict outcome^3^. Clinically, leveraging these measures is currently limited to invasive histological analysis using specific immunostainings of endothelial cells^4^. Non-invasive imaging approaches exist, such as infrared light physiology^5^, optical coherence tomography angiography^6^, perfusion weighted imaging^7^, and dynamic-contrast-enhance MRI^8^. These methods are subject to distinct limitations, including insufficient spatial resolution, restricted penetration depth, limited clinical applicability, and the requirement for sophisticated modelling to relate imaging-derived proxies to vascular architecture. Recent advances in shear wave technology have demonstrated the ability to overcome some of these limitations and to provide spatially localized maps of vascular architecture quantified through fractal measures^9^.

In light of these new opportunities, it is important to provide ground-truth information regarding vascular organization, here specifically for hepatocellular carcinoma (HCC). Several mathematical approaches exist to characterize vascular architecture^10–14^. Here, we focus on two different descriptors which are commonly used in the medical field: the fractal dimension $D_f$^15^ and the Hurst index *H*^16^. These descriptors have the appeal to quantify the spatial complexity of the vascular architecture in a single number, which can be used to compare different samples. In general, the fractality of the vascular system changes over scale^17^, and is conveniently quantifiable via the well-established box counting method^18^. While the box counting approach focuses on the complexity of the structures themselves, the Hurst index characterizes the distribution of the empty space between them. *H* describes the rate at which the distribution of lags (free space) between pairs of vessels increases/decreases as the size of the lag increases. Theoretically, *H* is directly related to the fractal dimension via $D_f=2-H$^19^. Thus, both concepts are complementary and operate at different scales to describe the same architectural properties. The theoretical relationship holds true only for ideal fractals, and we expect to observe deviations when characterizing tissue vasculature.

In the present study, we aim to answer the following fundamental questions: firstly, is the fractal dimension/Hurst index of the vascular architecture, derived from CD31 immunohistological staining using 3,3‘-diaminobenzidine (DAB) as chromogen (highlighting the vascular network), different between non-tumoral liver and tumoral liver tissues, and secondly, is tumoral and peri-tumoral vascular architecture impacted by the presence of vascular invasion, a key prognostic factor. If demonstrated effective, this fundamental knowledge would motivate further research that could enable the non-invasive quantification of these descriptors using novel imaging concepts^9,20,21^, and ultimately help for guiding therapies.

To address these questions, we present an image segmentation approach to isolate vascular structures from CD31 stained whole slide images.

Then, we demonstrate that the theoretical prediction between fractal dimension and Hurst index holds, when $D_f$ is quantified close to the smallest box size $(1\mu m)$, and *H* obtained from a fit to the lag distribution spawning approximately 1 order of magnitude in lag $(20-200 \mu m)$.

Subsequently, we investigate architectural changes in vascular organization at 3 different scales $(1, 16, 128 \mu m)$ via the fractal dimension, comparing tumoral, peritumoral, and non-tumoral tissues in cases with/without vascular invasion. Lastly, we shed light on which organizational properties in the vessel size distribution are responsible for the observed differences in fractality.

## Results

### Patient cohort

Our study consists of 29 tissue samples from a mixed cohort of patients, 21% female patients and 79% male patients, with a median age of 65 years (ranging from 43 to 89 years, see Table 1). This matches well the typical demographics of HCC patients. Regarding risk factors for chronic liver diseases, 31% had chronic hepatitis B, 38% had chronic hepatitis C, and 24.1% had a history of excessive alcohol consumption. Among them, 90% had cirrhosis. Tumor size ranged from 1.3 to 5.6 cm, with a median size of 3 cm. Additionally, 86% of HCCs were well or moderately differentiated, and 52% of tumors exhibited vascular invasion (Table 1).

### Validation of vessel segmentation algorithm

**Fig. 1:**
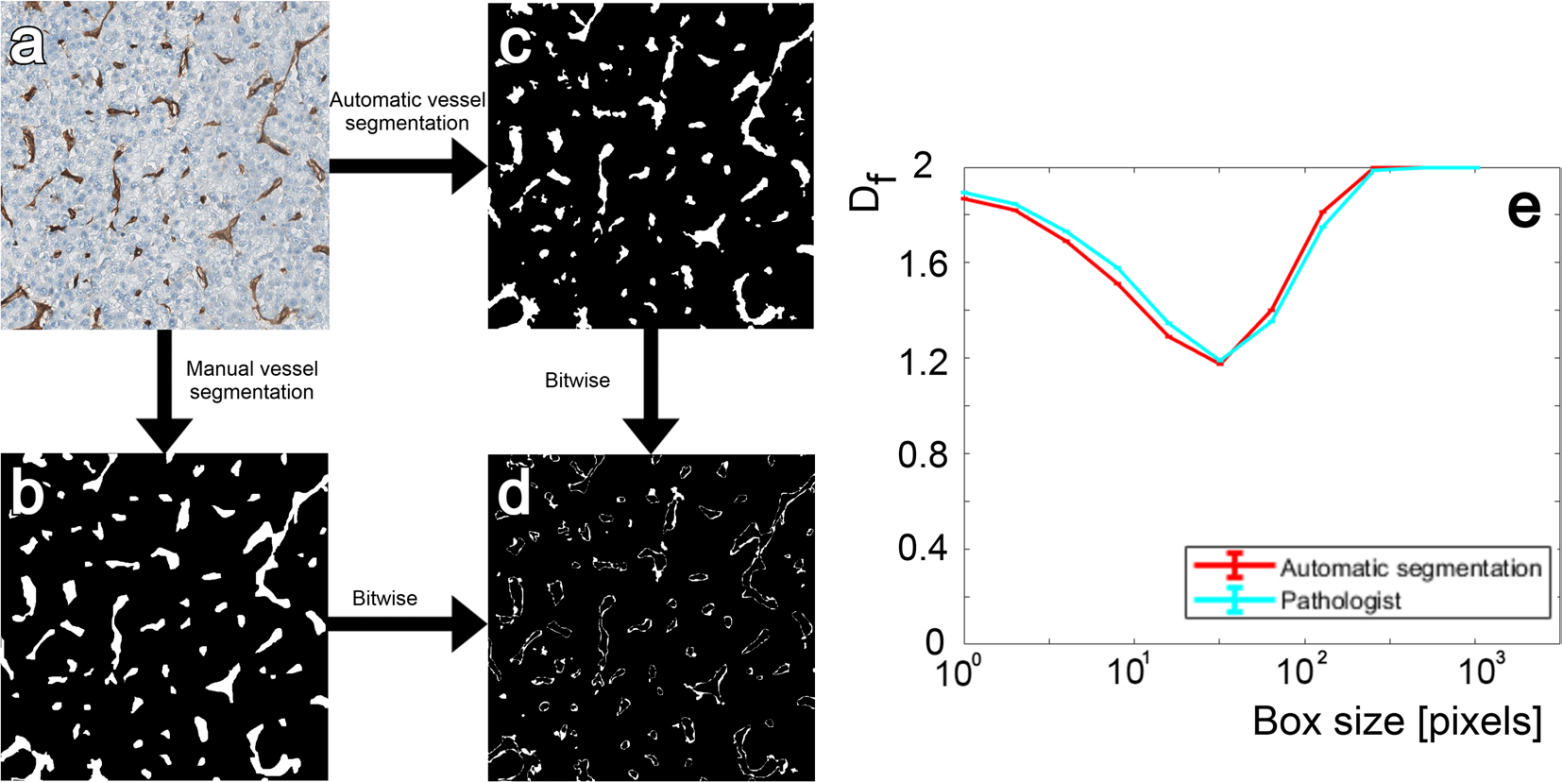
Vessel segmentation compared to pathologist manual segmentation. (**a**) CD31-stained tile of liver tissue. (**b**) Pathologist vessel segmentation of a. **(c)** Automatic segmentation generated by our algorithm with a dice score of 81% and a positive predictive value of 79%. (**d**) Bitwise comparison between (**b**) and (**c**). (**e**) The curve depicting the fractal dimension calculated by the local derivative of the box count on the Pathologist segmentation of (**a**) and the automatic segmentation of (**a**).

Figure 1a shows a representative CD31-immunostained liver section used for vessel analysis. The corresponding manual vessel segmentation of Fig. 1a performed by a pathologist with over 25 years of expertise in liver pathology is shown in Fig. 1b, while the automatic segmentation obtained using the proposed method is presented in Fig. 1c. Three tiles of CD31-immunostained slides were considered in regions containing 1024x1024 pixels $(\approx 512\,\times \,512 \mu m^2)$. On average, the algorithm achieved a Dice coefficient of 81% and a positive predictive value of 77% compared to the gold standard (pathologist annotation). As illustrated in Fig. 1d, the main discrepancies between manual and automatic segmentation are located at the edges of the vessels. In this context, these differences minimally impact the calculus (max deviation of 0.05) of the fractal dimension at any scale, as shown in Fig. 1e.

### Regional differences in fractality

**Fig. 2:**
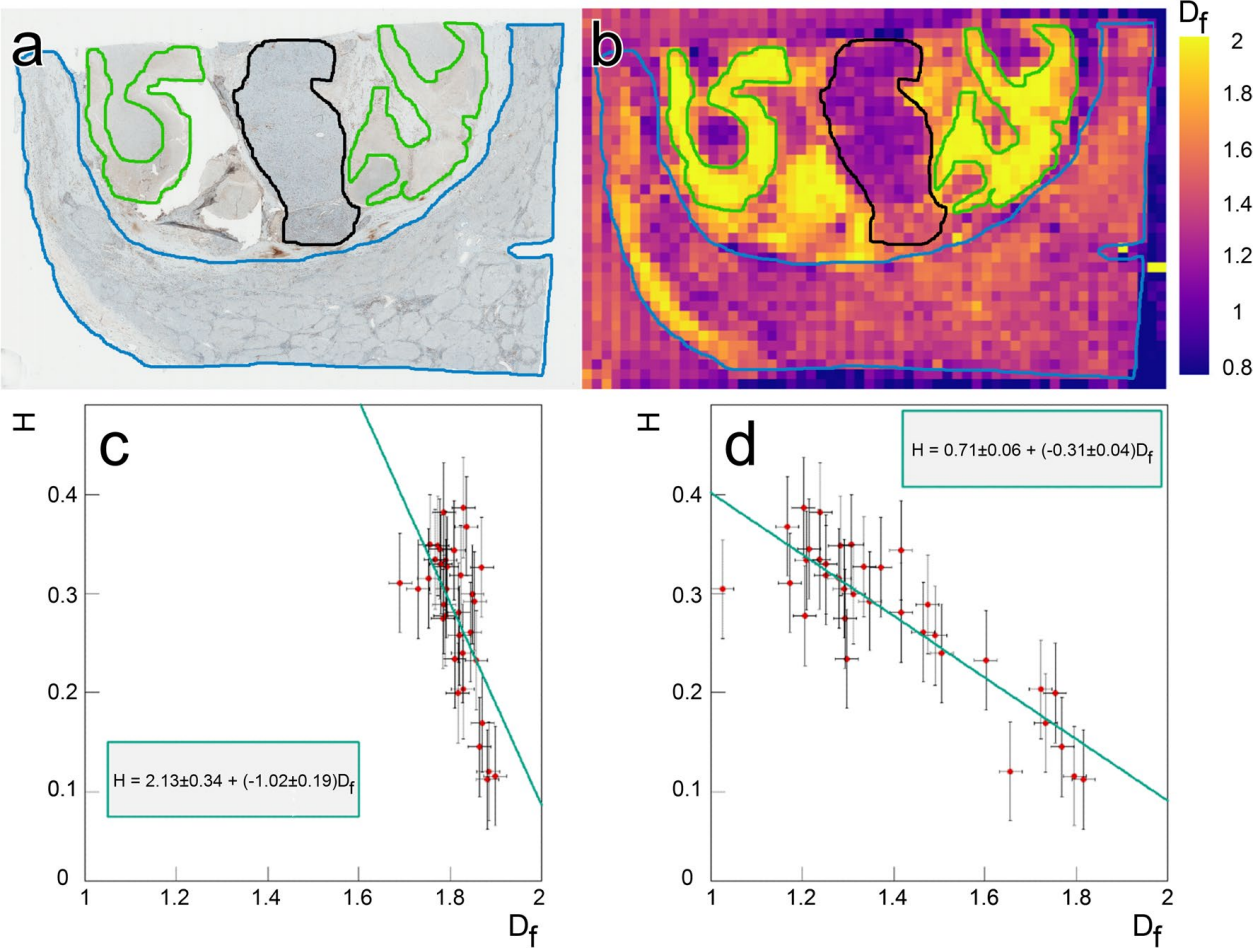
Comparison of histopathology with fractal dimension and Hurst index. (**a**) The original histopathological image with blue representing the peritumoral tissue, black indicating the tumor nodule, and green depicting necrotic tissue. (**b**) The original image reconstructed from the fractal dimension value of each 1024x1024 tile in the image. (**c**) Comparison of the Hurst index and the fractal dimension using a box size of 1 pixel. (**d**) Comparison of the Hurst index and the fractal dimension using a box size of 32 pixels.

Figure 2a shows a CD31 DAB immunostaining of a HCC (case 1) annotated, by a pathologist, into 3 regions (viable tumor area, necrotic area, and peritumoral area).

Importantly, this additional delineation of the necrotic area is shown for illustration purposes only: throughout the study, quantitative analyses were carried out exclusively on the three predefined regions–tumoral, peri-tumoral, and distant non-tumoral tissue–and necrotic patches were systematically excluded from all region-wise comparisons.

The corresponding image of the fractal dimension using a box size of 32 pixels is presented in Fig. 2b. Clearly, different anatomical regions exhibit very different mean fractal dimensions, with the tumor zone well delineated from its surrounding tissue. The vascular organization of the tumor area exhibited lower fractality compared to the peri-tumoral area. Because of the nature of CD31 DAB staining, the background within necrotic regions appears diffusely brown, closely resembling, similar to the color used to identify the endothelial cells lining the vessels. As a result, the segmentation algorithm interprets this uniform brown background as a continuous ”vessel-like” signal, causing the entire necrotic area to be segmented. This produces fractal dimension values close to 2 across all box sizes, as illustrated in this case and detailed in Supplementary Fig. 2. That confounding aspect has been considered in all further analysis by actively excluding necrotic patches from any zonal analysis. Mind that the presence of any remaining patches of dispersed necrosis as background in regions containing vessels is consequently leading to an artificial increase in fractality, and not a decrease. We also observe that regions of the image not containing tissue still exhibit non-zero fractal dimension values; this can be attributed to the presence of debris (see Supplementary Fig. 1). Fig. 2c,d show the correlation for multiple samples (non-tumoral and tumoral) between the fractal dimension calculated at two different box sizes (2, 32) and the lagtime quantified in the range from $20-200\mu m$, respectively. The expected theoretical relationship $H=2-D_f$ is found within errors for the smaller box size (Fig. 2c). Finding this theoretical relationship in our analysis is very reassuring and permits us to freely use either mathematical construct. Our analysis reveals that $D_f$ is taken at the specific scale of 32 pixel box size, where a linear relationship between *H* and $D_f$ can still be observed, but with a modified slope and DC. This originates from scale differences and the fact that vasculature does not represent an ideal fractal (Fig. 2d).

**Fig. 3:**
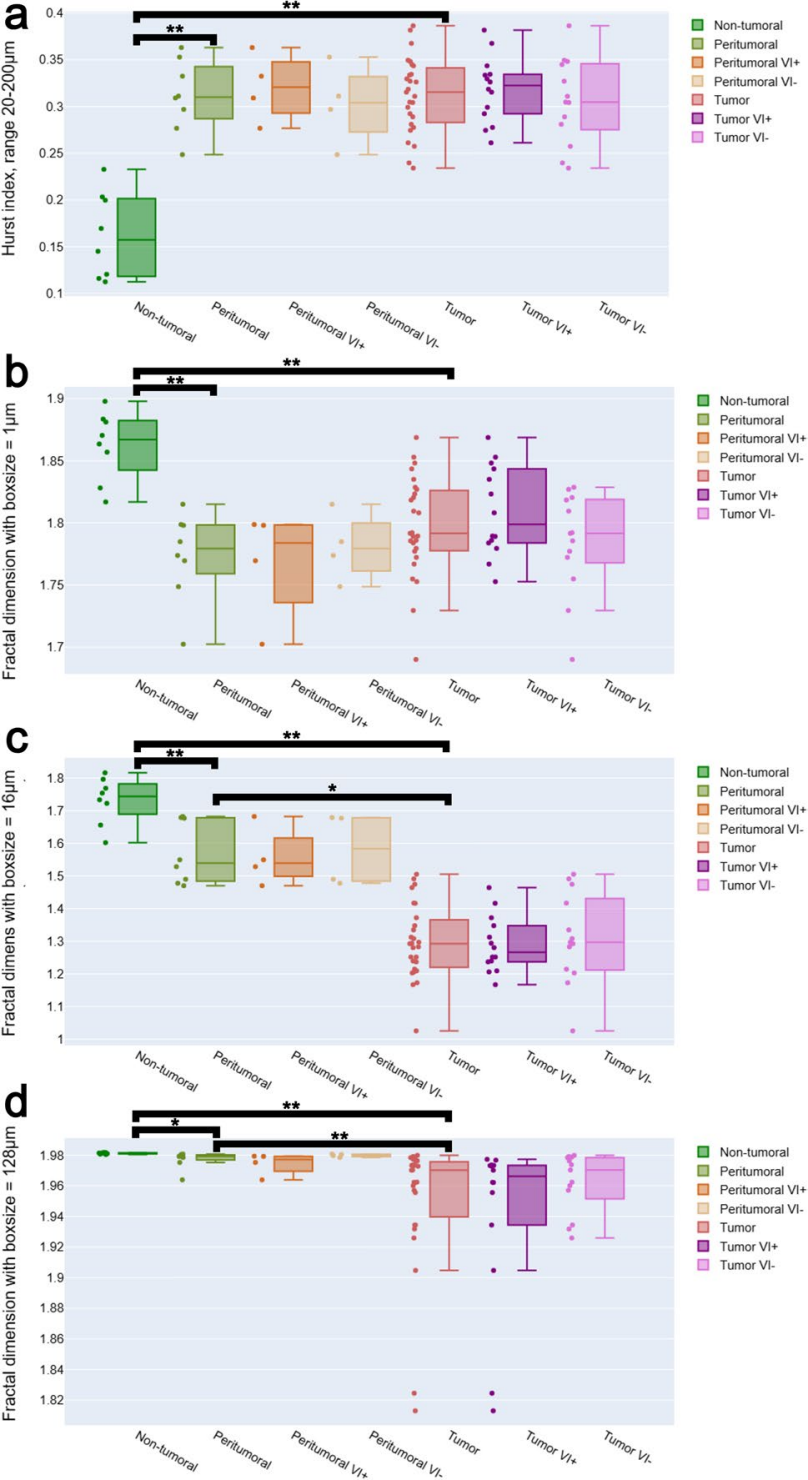
Tissue type a function of the fractal dimension/hurst index using different scales/box-size for the vascular network in 10,000x10,000-pixel tiles from histopathological data. The presence or absence of vascular invasion is indicated by VI+ and VI-, respectively. (**a**) Hurst-index using a scale of $20-200\mu m$. (**b**) The fractal dimension using a box size of $1 \mu m$ which verifies $H=2-D_f$. (**c**) The fractal dimension using a box size of $16 \mu m$. This is the scale where the difference between tumoral, peritumoral and non-tumoral is the most demarked. With $\overline{D_f^{Non-tumoral}} =1.29 \pm 0.02$, $\overline{D_f^{Peritumoral}} =1.54 \pm 0.03$, $\overline{D_f^{Non-tumoral}} =1.74 \pm 0.02$, $\overline{D_f^{Tumor VI+}} =1.27 \pm 0.02$, $\overline{D_f^{Tumor VI-}} =1.30 \pm 0.04$, $\overline{D_f^{Peritumoral VI+}} =1.54 \pm 0.04$ and $\overline{D_f^{Peritumoral VI-}} =1.58 \pm 0.05$. (**d**) The fractal dimension using a box size in the range of what H is calculated from, a box size of $128 \mu m$. Comparisons with* p* $< 0.001$ are marked with ** and those with* p* < 0.05 with *.

Figure 3 presents the distribution of the Hurst index (a) and the fractal dimension (b–d) across distinct histological zones: non-tumoral, peritumoral (± vascular invasion), and tumoral (± vascular invasion) regions. The Hurst index (panel a, calculated in the interval from $(20-200\mu m)$) shows a marked increase from non-tumoral tissue to peritumoral and tumoral regions, indicating a transition from smoother to more spatially correlated vascular patterns. Within tumoral areas, the presence or absence of vascular invasion (VI+/VI-) does not substantially alter the Hurst index.

The fractal dimension exhibits a scale-dependent behavior: it is lower in peritumoral and tumoral zones than in non-tumoral tissue already at small scales $(1\,\mu m)$, and this separation widens at mesoscopic scales $(16\,\mu m)$. At large scales $(128\,\mu m)$, non-tumoral tissue remains near space-filling $(\approx 2)$, whereas peritumoral and tumoral regions stay distinctly lower. Globally, the impact of VI+/VI- is not statistically significant at any scale, independent of the considered zone (for peritumoral $p = 0.79$ and tumorous $p = 0.33$). At the mesoscopic scale, we find a clear drop in fractal dimension for the tumour region compared to the surrounding peritumoral region. Overall, tumor-associated vasculature shows higher spatial correlation (higher *H*) but reduced space-filling complexity (lower $D_f$) across scales, most pronounced at intermediate scales, while vascular invasion is not impacting fractality at any scale.

Consequently, from our data we can conclude that the vascular invasion status does not statistically impact the spatial architecture of the vasculature, neither within the tumor $(p = 0.33)$ nor in the peri-tumoral zone $(p = 0.79)$. However, we observe that the presence or absence of a tumor itself does impact, with high statistical significance, the vascular architecture $(p < 0.001)$. Statistical significance is also observed when comparing peri-tumoral tissue to non-tumoral tissue $(p < 0.001)$ and tumorous tissue $(p = 0.002)$ at the mescoscopic scale.

### Geometrical basis of fractality differences in vascular organization

**Fig. 4:**
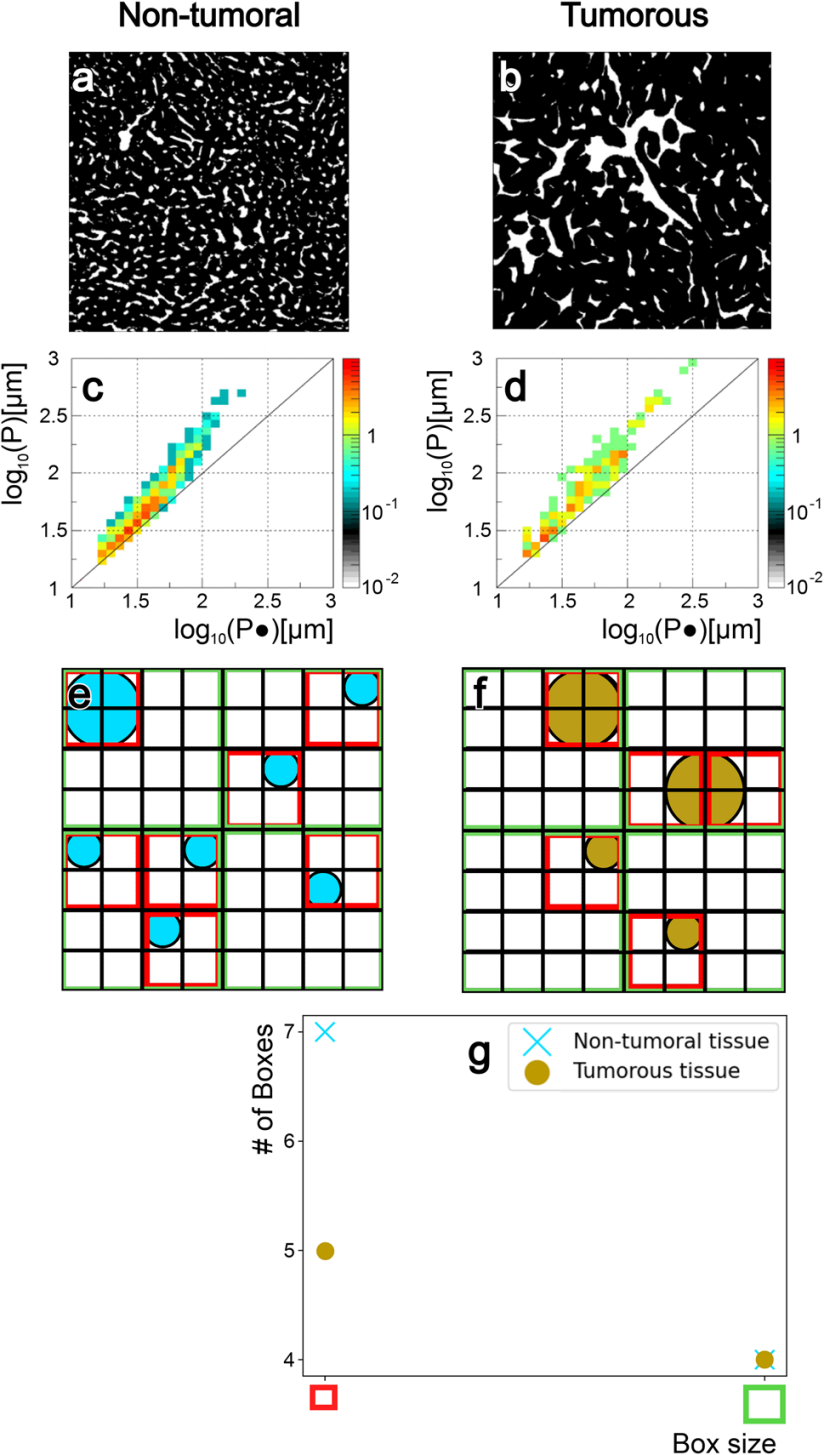
Why does vessel shape impact the fractal dimension? (**a**) A tile of non-tumoral tissue processed by our vessel extraction algorithm. (**b**) A tile of tumorous tissue processed by our vessel extraction algorithm. (**c**) Density map of vessel contour size in relation to the contour size of a circle with the same area as the vessel for non-tumoral tissue. (**d**) Density map of vessel contour size in relation to the contour size of a circle with the same area as the vessel for tumorous tissue. (**e**) Example of a vascular network for non-tumoral tissue, comprising 6 small vessels and 1 large vessel. (**f**) Example of a vascular network for tumorous tissue, comprising 2 small and 2 large vessels. (**g**) Box count plot for the vascular networks shown in e and f. For intermediate box sizes (4 units, green; 2 units, red), the slope difference reproduces the expected trend in fractal dimension ($D_f$), with non-tumoral tissue showing a higher $D_f$ due to its more heterogeneous distribution enriched in smaller vessels, whereas tumorous tissue displays a more homogeneous distribution across vessel sizes.

The geometric origin of the difference in fractality between vessel organization in tumoral and non-tumoral liver tissue is illustrated by their density distributions within the perimeter plane $P - P\cdot$ (Fig. 4a-d). As mentioned beforehand, the $45^\circ$ line represents the limit below which no entry can occur. For small perimeters ($P<101.75=56mm$), most vessels cluster along this line, indicating predominantly round shapes (Fig. 4c,d). At larger perimeters ($P>56mm$), the distribution deviates from the $45^\circ$ line, showing that larger vessels become more elongated. While the total vessel areas are comparable between non-tumoral and tumoral tissues ($\approx 13\%$), their density distributions differ significantly. Non-tumoral tissue shows a steep increase in density toward small, circular vessels, whereas tumoral tissue displays a more homogeneous distribution. This shift explains the lower fractal dimension observed in tumoral tissue. As illustrated in the toy-model examples (Fig. 4e–g), a non-tumoral configuration with one large and several small vessels yields a higher $D_f$ than a tumoral configuration with fewer small vessels. Using the box-counting method, more boxes are required at smaller scales to cover the numerous small vessels, resulting in a steeper slope and thus a higher estimated fractal dimension.

## Discussion

Reliable vessel segmentation is essential for accurately quantifying vascular organization. Our proposed method achieved Dice scores against expert manual segmentation that are comparable to those reported in recent literature^22^. The absence of a gold standard for automatic segmentation of vessels on CD31 DAB immunostained tissue led us to rely on manual segmentation to quantify the performance of our approach. It should be noted that manual segmentation performed by a pathologist inevitably introduces a certain degree of variability. As illustrated in Fig. 1d, most discrepancies between manual and automatic segmentations occur along vessel boundaries. Such minor differences are expected, given the inherent difficulty of delineating vessel contours with high precision. In the context of our analysis, we focused on mathematical descriptors of the vascular network, specifically the fractal dimension and the Hurst index. Here, minor inconsistencies in vessel boundaries have a negligible impact on the results, as seen when comparing the calculation of the fractal dimension between the pathologist’s segmentation and our automatic segmentation in (Fig. 1e). This demonstrates that, despite small boundary inconsistencies, our automatic segmentation provides a reliable mask for quantifying vascular architecture.

In contrast to the highly organized microstructure of non-tumoral vascular networks, pathological processes often drive the vasculature toward anomalous architectures that can be distinctly characterized through histopathology. Capturing these alterations non-invasively would provide crucial pathophysiological biomarkers for disease diagnosis and treatment monitoring. Recent advances suggest that the fractal dimension (or equivalently, the Hurst index) can be quantified through non-invasive wave-scattering experiments^9^. In view of this opportunity, it is of fundamental importance to evaluate–using histopathology as the ground truth–whether changes in vascular fractality serve as proxies for underlying pathologies, such as the presence or absence of vascular invasion, a key prognostic factor in HCCs.

The theoretical relation $D_f = 2 - H$ is exact only for ideal fractals that are self-similar across all scales. However, biological vasculature is not a perfect fractal but exhibits scale-dependent organization. This explains why we observe correspondence between $D_f$ and *H* only within a restricted interval (Fig. 2c, d). In the box-counting analysis, the most discriminant scaling regime for distinguishing tissue types occurs at $8-32$ pixels ($\approx 4-16 \mu m$) (Fig. 3c), which corresponds to the characteristic size of small vessels and sinusoidal branching. At this scale, the fractal dimension captures how vessels fill space locally. In contrast, the Hurst index is obtained from the lag distribution of free space between vessels at $20-200 \mu m$ (Fig. 3a), corresponding to intervascular spacing at the lobular level. Interestingly, when the fractal dimension is evaluated using a box size of $128 \mu m$ (Fig. 3d), midway within the range over which *H* is computed, the same trends are observed: peritumoral tissue becomes indistinguishable from tumoral tissue, as seen for *H*. This indicates that when both descriptors are probed at comparable scales, they capture the same underlying spatial organization. At smaller scales ($<20 \mu m$), the limited amount of data leads to unreliable estimation of the Hurst index; therefore, we exclude this range from the calculation.

Although the fractal dimension and the Hurst index capture different aspects of spatial organization, they are mathematically and structurally linked: the way vessels occupy tissue at the micron scale constrains how voids are distributed at the mesoscopic scale. Where this structural–complementary coupling is strong, the theoretical identity $D_f = 2 - H$ emerges; where it weakens (very small scales dominated by vessel wall thickness, or very large scales dominated by tissue boundaries), deviations are expected. This behavior is consistent with the well-known multifractality of biological networks, where different scaling exponents govern different structural regimes^17–19,23^.

It is particularly intriguing to observe a clear-cut difference in $D_f$ between non-tumoral liver parenchyma and tumorous tissue. This difference was highly significant $(p < 0.001)$, with minimal overlap, and demonstrated the fundamental divergence in vessel architecture. The subsequent analysis of the probability distribution of vessel shapes traced this difference in fractality back to the observation that, in normal tissue, there were more small, circular-shaped vessels, whereas in tumorous tissue, the density distribution of vessel shapes was more homogeneous across all sizes. This finding aligns with previous observations that quantified vessel distributions within liver lesions via photoacoustic microscopy^24^.

From histopathology, it is known that sinusoids splice into smaller vessels during the development of fibrosis and cirrhosis^25^.This represents an opportunity for novel non-invasive methods sensitive to vascular architecture^9^ to probe this process and potentially quantify the degree of vessel splicing.

Furthermore, it is reassuring that the peritumoral zone exhibited values of $D_f$ that lie between those measured for non-tumoral tissue and those quantified for the tumor zone. This demonstrates that the presence of the tumor exerts an influence on its surrounding tissue, and that this biochemical and biomechanical impact leads to modifications in vessel organization. As a result, imaging biomarkers that are sensitive to vessel fractality will have the potential to gauge the extent of the impacted zone^9^. Moreover, no statistically significant association was observed between vessel fractality and the vascular invasion status, either within the tumor zone ($p = 0.33$) or within the peritumoral zone ($p = 0.79$). While this might initially appear disappointing, it likely reflects the biological reality: vascular invasion represents the spread of cancer cells from the tumor nodule into adjacent microvessels, which is indicative of the tumor’s metastatic potential. This condition, however, does not directly alter the vascular architecture, as illustrated in Fig. 3.

There are several limitations to our study. Firstly, necrotic regions exhibited elevated random CD31 staining (see Supplementary Fig. 2), leading to an artificial increase in fractality. This was visible within the two distinct zones marked in Fig. 2a,b. Although we took care to avoid regions with patchy necrosis in our analysis, its presence would increase the observed value of the fractal dimension within the tumor zone.

Furthermore, some non-tumoral tissue zones exhibited low fractality. This fluctuation was generally due to failed immunostaining, unsuccessful vessel extraction, or tears in the sample. In regions of the slide where tissue was absent (Fig. 2a), the observed random fractality can be attributed to debris particles, which introduce noise into the analysis (see Supplementary Fig. 1).

Taken together, our results show that the vascular architecture, quantified via its fractal dimension, differs clearly between the central regions of HCCs, their peritumoral zones, and normal liver parenchyma. This difference can be traced back to variations in vessel shape distribution (Fig. 4), with tumor regions exhibiting fewer small, round-shaped vessels than their non-tumorous counterparts. We further show that vascular invasion does not affect vascular fractality. These promising findings motivate further investigation into non-invasive approaches capable of quantifying fractality, thereby adding valuable diagnostic information.

## Methods

### Ethical statement

All methods were carried out in accordance with local guidelines and regulations and the declaration of Helsinki. Written informed consent was obtained from all patients in accordance with French legislation. This study was registered with the National Commission Informatics and Liberty (CNIL) and received approval from the local ethics committee (CEERB PARIS NORD IRB00006477, Protocol Number CER-2022-168) and data for the study had been anonymized.

### Histological assessment and sample preparation

Vascular architecture was assessed by CD31 immunostaining in liver surgical specimens from 29 patients with HCCs collected in Beaujon hospital. The processed dataset consisted of 35 whole slide CD31 images obtained from representative formalin-fixed paraffin-embedded tissue sections. Of the 35 slides, 27 were derived from the tumors with their surrounding peritumoral areas, while 8 samples were derived from non-tumoral liver distant from the tumors. Detailed histological analysis (Hematein & Eosin (H&E) staining) of HCC informed about the degree of tumor differentiation according to the WHO classification, and presence or absence of vascular invasion (VI+/VI-, respectively). Additionally, histological analysis provided the status of the background liver parenchyma (presence or absence of cirrhosis). CD31 immunostaining (clone JC/70A, Santa Clara reference MB82301, Dako, dilution 1:200) was performed on a $3 \mu m$ thick cut-section using an automated immunohistochemical stainer according to the manufacturer’s guidelines (streptavidin-peroxidase protocol, Benchmark Ventana), using 3,3‘-diaminobenzidine (DAB) as chromogen. For each sample, tumoral and peri-tumoral tissue regions of interest were annotated by a pathologist.

### Fractal analysis

**Fig. 5:**
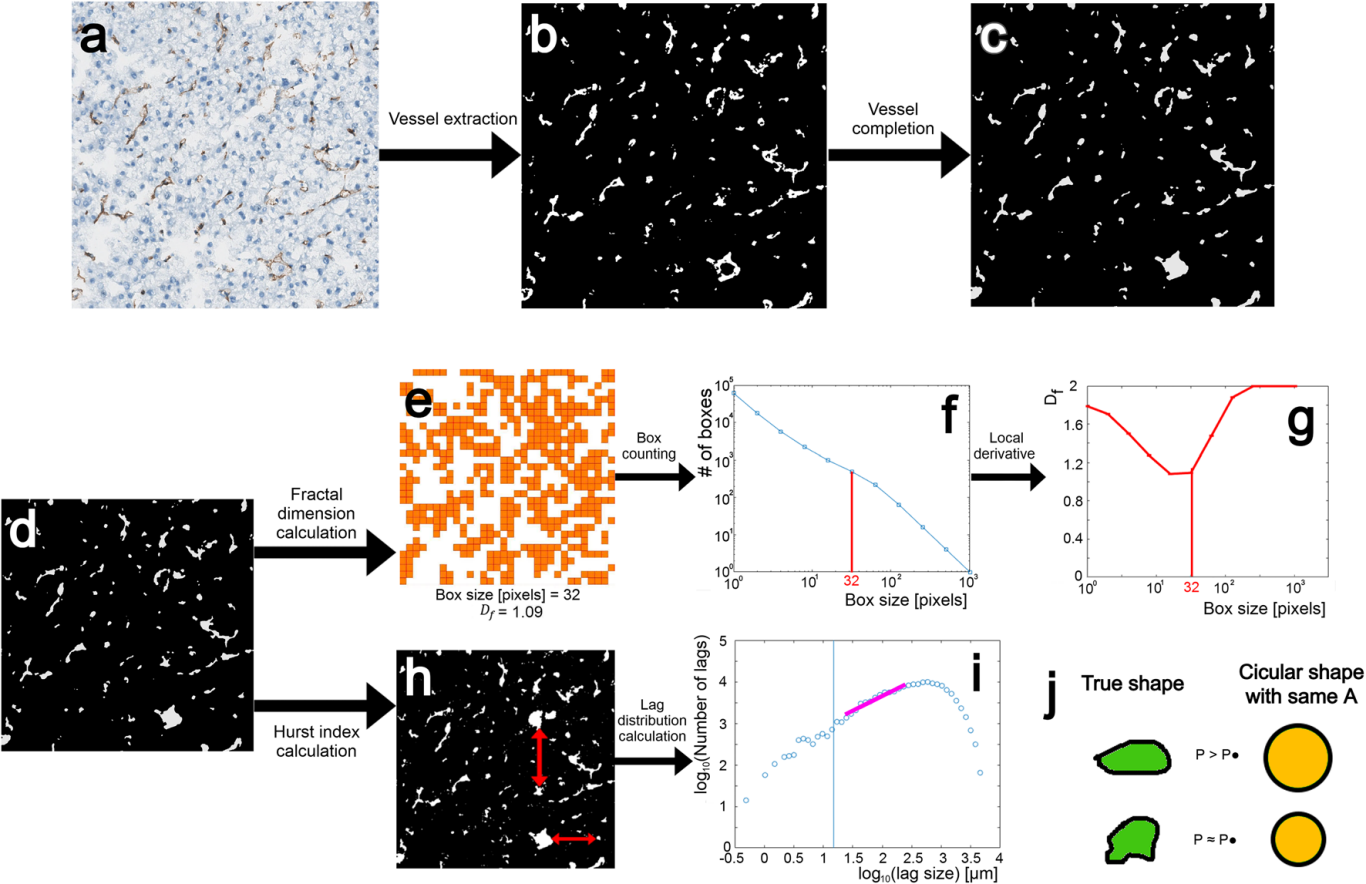
Segmentation and Fractal Analysis. (**a**) 1024x1024 tile extracted from the larger histopathological image. (**b**) The same tile after the vessel extraction step. (**c**,** d**) The tile after vessel completion. (**e**) The tile with vessels boxed using a 32-pixel box size. (**f**) Evolution of the number of boxes required to cover vessels based on box size. (**g**) The curve depicting the fractal dimension calculated by the local derivative of the box count with a value of 1.16 when using a box size of 32. (**h**) Tile with vessels extracted; red arrows indicate free space between vessels, termed as a lag. (**i**) Distribution of the number of lags for each lag size on a logarithmic scale. The pink line represents the distribution curve between 20 microns and 200 microns. The slope of the curve corresponds to the Hurst index in this case $2H = 0.73498$. (**i**) Comparison between the true shape of vessels and their theoretical circular shape with the same area as the initial vessel.

Fractal analysis such as the box counting method or the quantification of the Hurst index via the lag distribution necessitates to first segment the vessels from the whole slide CD31 stained images, with a spatial scale of $1 voxel = 0.5 \mu m$, and thus generate a binary image. Our segmentation approach consists of three steps: tile extraction, vessel extraction and noise reduction, and finally vessel surface completion, which are detailed in the following (Fig. 5a–c):

*Step 1* Original images with typical sizes of 20000x30000 pixels (equivalent to $1x1.5cm^2$) were subdivided into tiles of 1024x1024 pixels (512x512 $microns^2$) to obtain subregions which are likely to correspond to one individual tissue type, i.e., non-tumoral liver tissue, tumorous region, cirrhotic liver parenchyma, or peritumoral region. Certainly, this method fails when the tile is located just at a transition zone. The attribution of a tile to a specific tissue class was determined by the corresponding histopathological annotation of area in the whole slide the tile originated. This step enabled us to link vascular characteristics to pathological properties (Fig. 5a).

*Step 2* Vessel contour extraction was based on digital images from CD31 DAB immunohistochemical stainings, in which appear brown due to the DAB chromogen used. We start by eliminating all non-brown pixels. To achieve this, we set all pixels with a dominant green or blue channel to white. Additionally, we converted the image into the HSV (hue, saturation, and value) color space; we set all pixels with a hue value out of the range 20-40, which brown’s hue value range, to white. To further eliminate the remaining background, the image was converted back to the RGB color space and extracted the blue channel from the image. From this blue channel, pixels with intensities greater than 220 (on a scale of 0-255) were set to white, while those lower value intensities were set to black, resulting in a binary image. Subsequently, we invert the colors so that vessels appear white, and the background appears black.

To remove the brown noise caused by small non-vessel components marked by the DAB chromogen used, we assess the noise level by examining the number of pixels in the tile with a red value above 210 (out of 256 maximum). If more than half of these pixels meet this criterion, we apply a stronger median blur filter with a radius of 15 pixels to help eliminate the noise. Otherwise, we apply a standard median blur with a radius of 5 pixels (Fig. 5b).

*Step 3* To convey the entire space occupied by the vessels, we need to complete the vessel’s entire surface and not just the contours. In this step we apply two dilation operations to close the vessel contours. We utilize the find contours function from OpenCV to fill in the gaps in the white circles (representing vessels in our case). Subsequently, we perform two iterations of erosion to restore the vessels to their original sizes (Fig. 5**c**).

Tiles containing images of vessels in binary contrast (Fig. 5d) are subsequently used for the two proposed fractal analysis approaches for vessel architecture characterization, i.e. quantification of the fractal dimension via box counting using a fixed grid, and estimation of the Hurst index via the lag distribution.

Fractal dimension quantification: Fig. 5e shows the coverage of all vessels within the example tile via boxes of size 32x32 pixels (orange boxes), with its corresponding number required for the calculation of $D_f$. Repetition of this process for different box sizes yields the classical box-counting curve, i.e., number of boxes needed to cover the structure as a function of box size (Fig. 5f), with the negative of its local derivative on a log-log plot representing $D_f$ at that scale (Fig. 5g). When attaining box sizes which cover the entire image or the individual pixel we approach values close to 2. Our results show that the lowest value of $D_f$ in our images is typically obtained for a box size of 32 pixels (16 microns). The variation of the fractal dimension with scale has been observed previously^23^ and is an expression of the fact that vasculature does not represent a perfect mathematical fractal extending indefinetly over all scales.

Hurst index quantification: Fig. 5h shows as an example two lags, i.e. free distances between obstacles. The corrsponding frequency distribution of all lags (calculated in horizontal and vertical direction) on a log-log scale is presented in Fig. 5i, with the slope representing 2*H*. Typically, surface roughnesses of the structures are probed at very small lags ($<15 \mu m$) with a corresponding value of $H\approx 1$, indicative for persistent fractality. The lag range of our interest starts beyond that regime with values of $H\in [0,0.5]$ which is the so-called anti-persistent regime characterizing a rough material where longer lags are followed by shorter. Ultimately, the lag distribution falls off for larger lags due to shielding effects. The range in which fitting is performed in our analysis for extracting *H* was therefore chosen from $20\mu m$ until $200\mu m$. To ensure sufficient data points for a robust Hurst index estimation, the analysis region was extended beyond the standard 1024x1024 pixel tile size. However, each extended region was restricted to a single histopathologically annotated tissue class.

Differences in vascular fractality are explored for non-tumoral liver tissue, the peritumoral zone, and the tumor region itself. Additionally, depending on the histopathological results, the tumor class is subdivided into those with/out vascular invasion. To relate the differences in fractal dimension to some tangible geometrical property, we furthermore investigate the repartition of vessels in terms of their circumference and their deviation from a perfect circle. Figure 5j shows the proposed concept: the vessel segmentation algorithm provides us for each vessel with its corresponding surface *A* and its perimeter *P*. We can now construct a hypothetical corresponding vessel with the same surface *A*, but circularly shaped. It would attain the smallest possible perimeter $P \cdot$ of all 2D shapes which exhibit the same surface *A*, i.e. $P\ge P \cdot$ always holds. Investigating the density distribution of vessels in this perimeter plane $P - P \cdot$ allows us to understand differences between tumor and non-tumor vasculature in terms of shape-density distribution.

### Statistical analysis

Statistical significance was assessed using the Mann-Whitney U test. Results with $0.001 \le p < 0.05$ were considered statistically significant, those with $p < 0.001$ were considered highly significant and those with $p > 0.1$ were considered not statistically significant.

**Table 1:** Main clinic characteristics of the patients included in the study

Patient	Age	Gender	Hep. B	Hep. C	EAC	Cirrhosis	HCC Size (cm)	VI
1	62	Male	Yes	No	Yes	Yes	2.7	No
2	53	Male	No	Yes	Yes	Yes	3	Yes
3	43	Female	Yes	No	No	No	3.5	No
4	71	Male	No	No	No	No	4.5	No
5	61	Male	No	No	Yes	Yes	1.8	No
6	71	Male	No	No	Yes	Yes	5	No
7	64	Male	No	No	Yes	Yes	4.5	No
8	70	Female	No	No	No	No	3	No
9	58	Male	No	Yes	No	Yes	3	Yes
10	43	Male	Yes	No	No	Yes	3.5	Yes
11	57	Female	No	Yes	No	Yes	1.5	Yes
12	69	Female	Yes	No	No	Yes	2.3	No
13	54	Male	No	Yes	No	Yes	3	Yes
14	59	Male	No	No	No	Yes	3	Yes
15	66	Female	No	Yes	No	Yes	4.8	Yes
16	78	Male	No	No	No	Yes	4	No
17	65	Male	No	No	No	Yes	2.5	No
18	73	Male	No	No	Yes	Yes	4	Yes
19	70	Male	No	No	No	Yes	3.8	Yes
20	66	Female	No	Yes	No	Yes	3	No
21	48	Male	No	No	No	Yes	5.6	Yes
22	55	Male	No	Yes	Yes	Yes	2.6	Yes
23	72	Male	No	Yes	No	Yes	1.3	No
24	73	Male	No	Yes	No	Yes	2.5	Yes
25	70	Male	No	Yes	No	Yes	3.2	No
26	75	Male	No	No	No	Yes	3	Yes
27	45	Male	No	No	No	Yes	4	No
28	55	Male	No	No	No	Yes	2.8	Yes
29	89	Male	No	Yes	No	Yes	3	No

Vascular invasion noted as ”VI”. Excessive alcohol consumption noted as ”EAC”. Hepatitis noted as ”Hep”. Patient 22 is currently undergoing transcatheter arterial chemoembolization (TACE).

## Supplementary Information

Supplementary Information.

## Data Availability

The data supporting the findings of this study are available from the corresponding author upon reasonable request. Due to privacy concerns, the dataset is not publicly archived. Access to the data will be provided in compliance with institutional guidelines and applicable regulations to ensure individual privacy is protected.
