# Supplementary material for: Fractal analysis of hepatocellular carcinoma vasculature shows regional differences independent of vascular invasion
Source: Sci Rep. 2026 Feb 25;16:11049. doi: 10.1038/s41598-026-38580-x (PMC13043768; doi:10.1038/s41598-026-38580-x)
Supplement: Supplementary file 1 — Supplementary Information. [file 41598_2026_38580_MOESM1_ESM.pdf]

Supplementry information

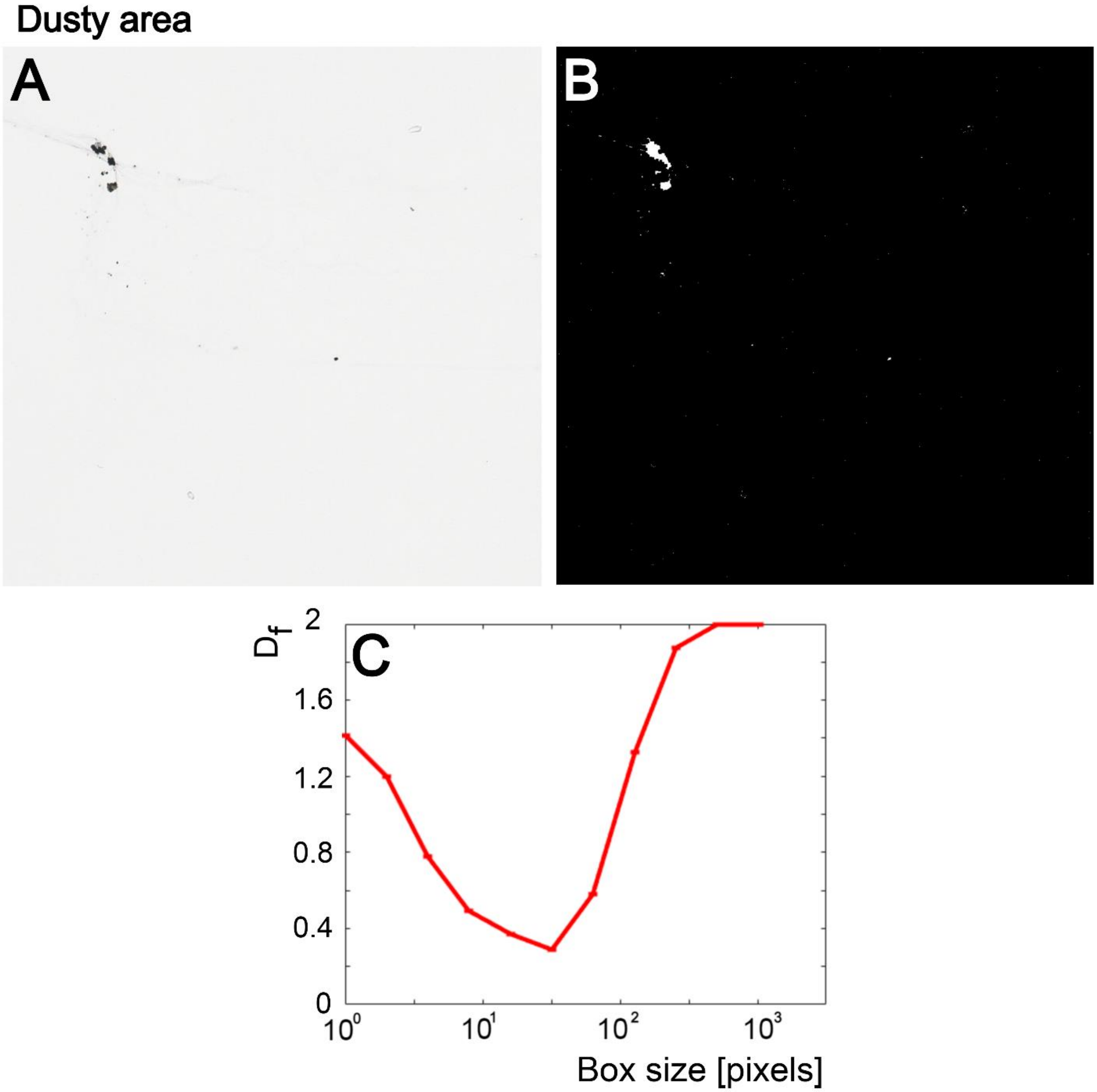

Supplementary figure 1 An empty tile containing debris was processed using the vessel segmentation pipeline, resulting in an abnormal fractal dimension. ***(a)*** *Tile before processing.* ***(b)*** *Tile after vessel extraction, with debris mistakenly identified as vessels.* ***(c)*** *Fractal dimension curve corresponding to (b).*

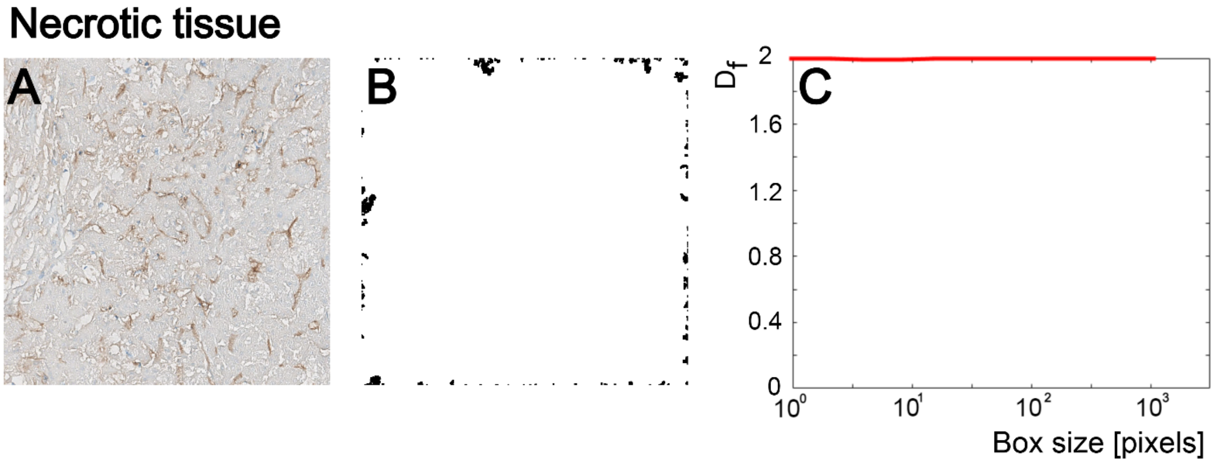

Supplementary figure 2 A tile from necrotic tissue was processed using the vessel segmentation pipeline, resulting in an abnormal fractal dimension. ***(a)*** *Tile before processing.* ***(b)*** *Tile after vessel extraction, with debris mistakenly identified as vessels.* ***(c)*** *Fractal dimension curve corresponding to (c).*

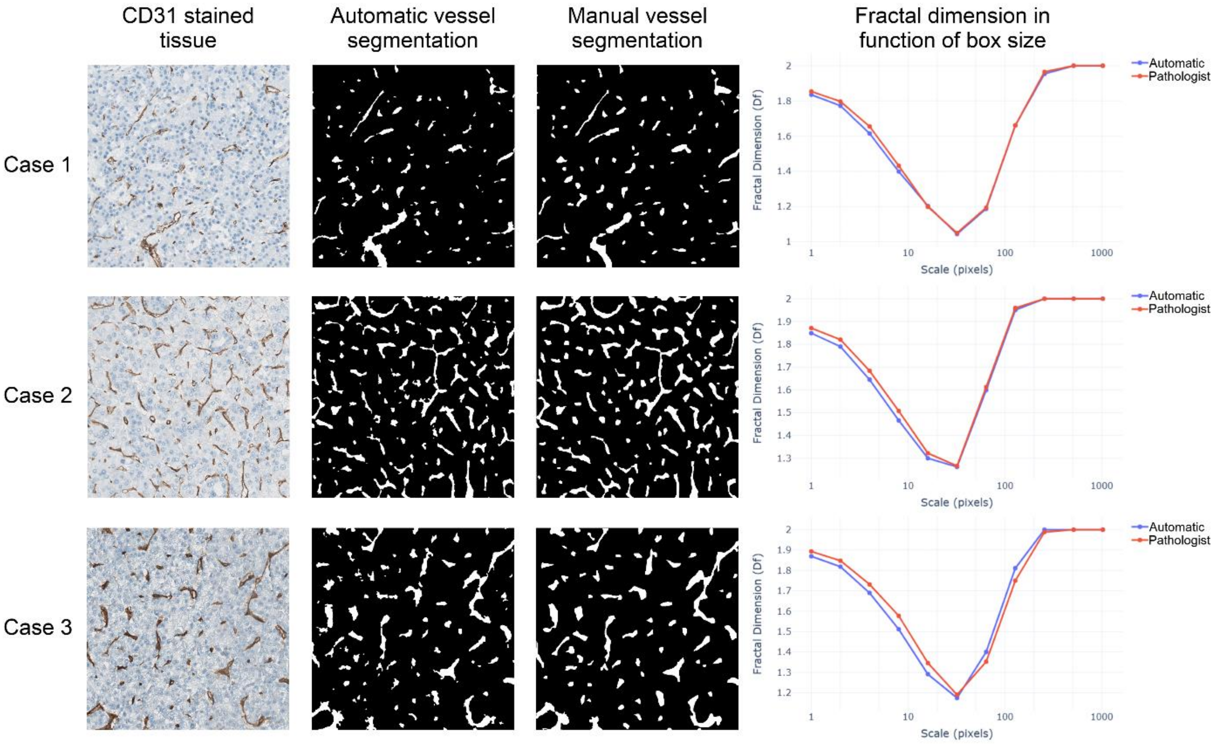

Supplementary figure 3 comparison of the fractal dimension in automatic segmentation versus manual vessel segmentation. The dice (automatic segmentation on manual segmentation) scores are as follows: case 1 = 0.79, case 2 = 0.83 and case 3 = 0.81. The PPV scores are as follows: case 1 = 0.75, case 2 = 0.78 and case 3 = 0.79.

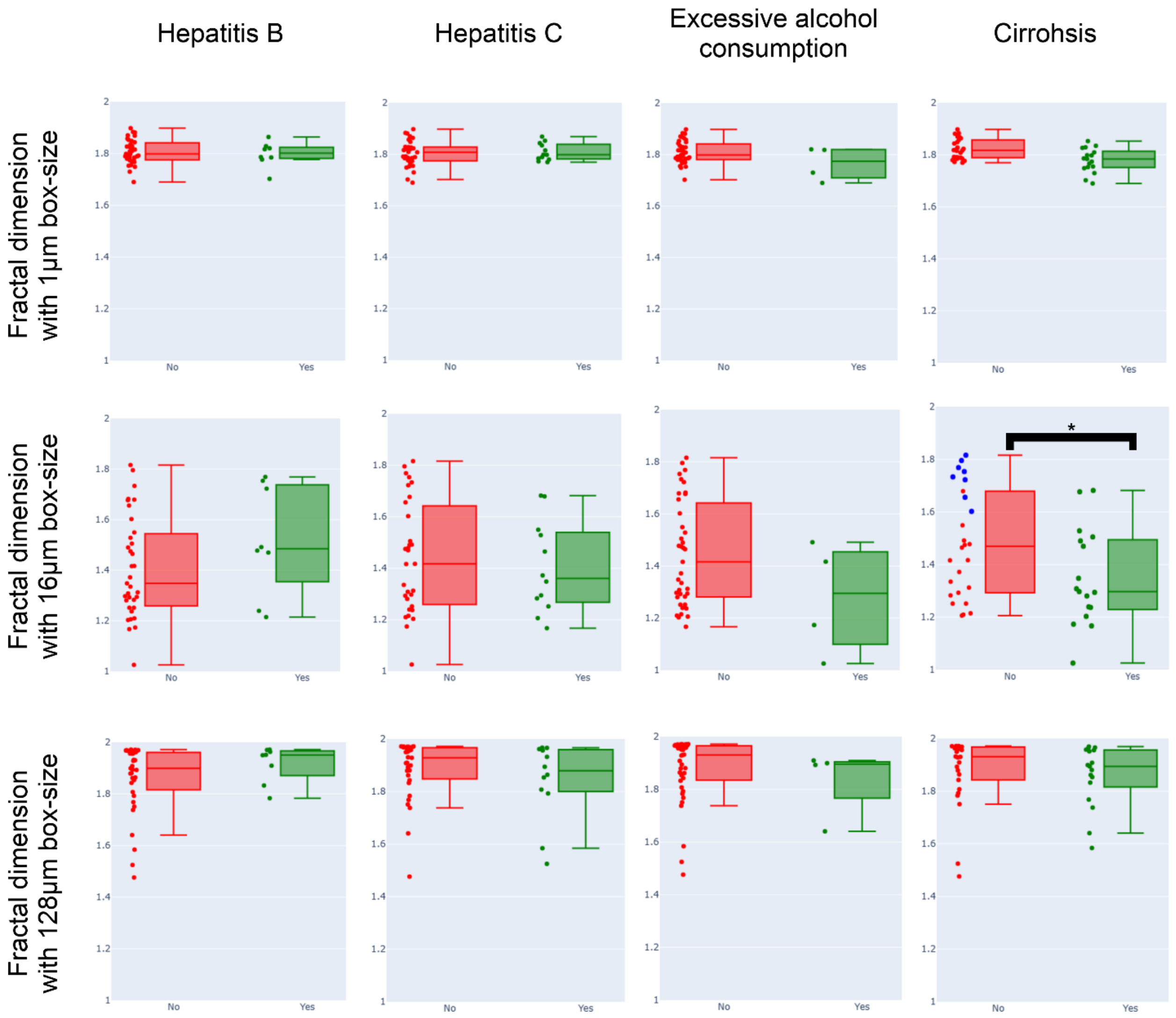

*Supplementary figure 4 Fractal dimension as a function of patient disease status. * denotes statistical significance ($p < 0.05$). Blue dots highlight the subset of non-tumoral samples in cirrhosis at the 16 µm box size.*

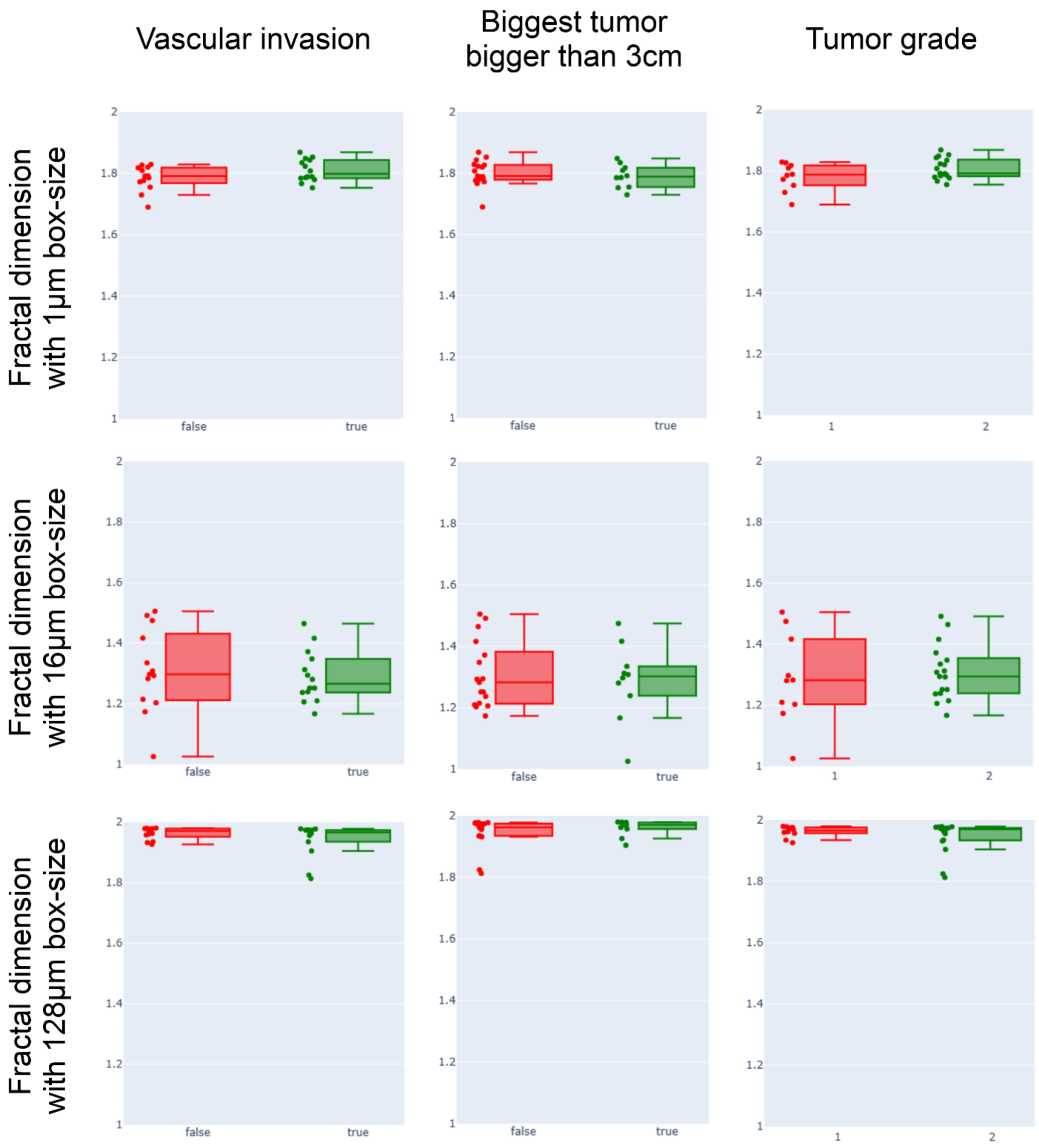

Supplementary figure 5 Fractal dimension as a function of physiological factors

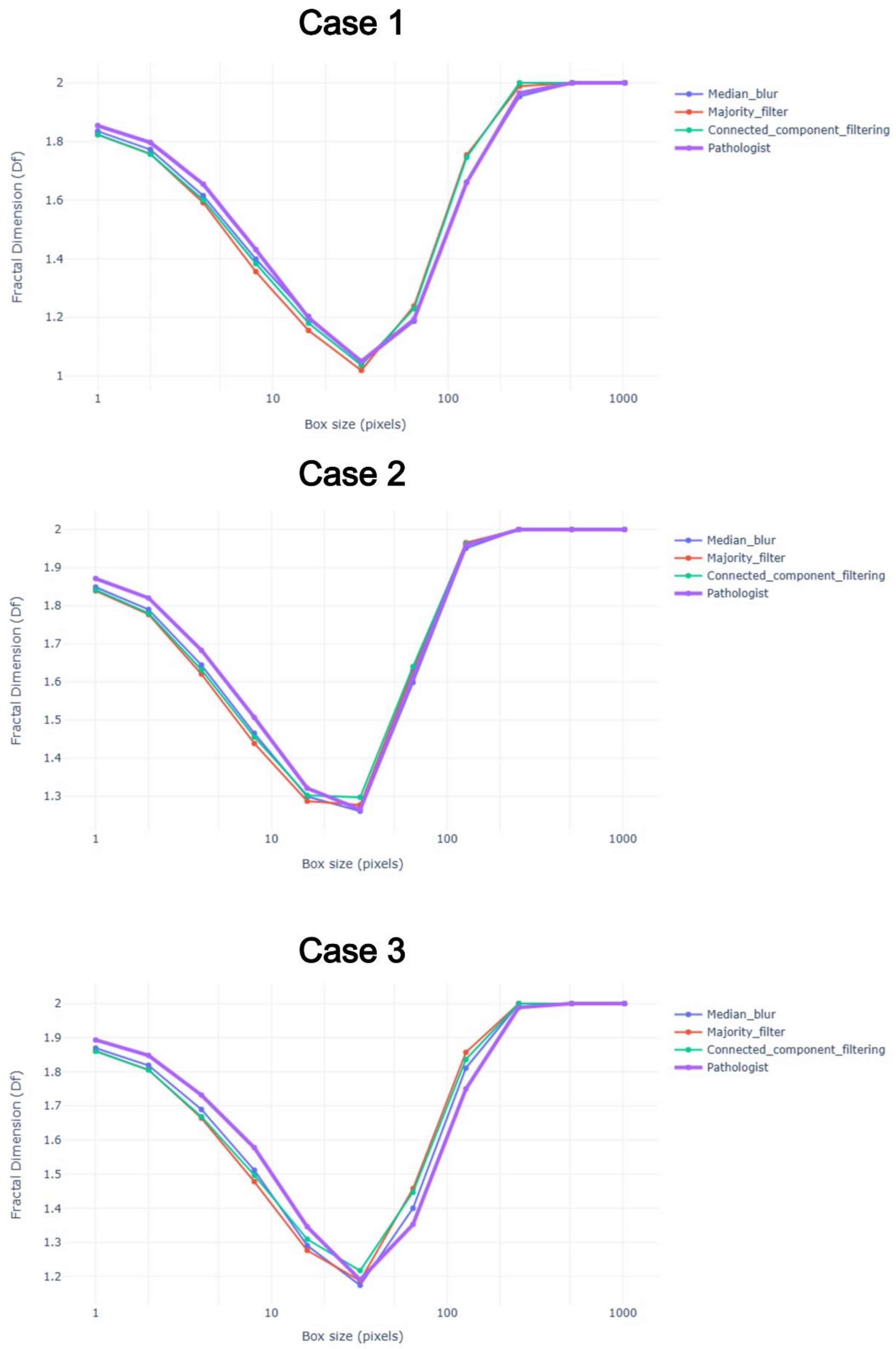

Supplementary figure 6 Fractal dimension (Df) as a function of box size for multiple cases (Cases 1–3 shown in Supplementary Fig. 3), computed across box sizes ranging from 1 to 1024 pixels. All Df values were obtained using the method described in the Methods section, with the only modification being the replacement of the median-blur filter by

alternative filters (majority filter and connected-component filtering), each applied with an equivalent kernel size. Results are compared with the fractal dimension computed from the pathologist's annotation of the same tile. Among the automatic methods, the median blur produces a Df curve that most closely matches the pathologist's reference. Dice scores show only minor variations across cases: Case 1 (Median_blur: 0.79; Majority_filter: 0.79; Connected_component_filtering: 0.79), Case 2 (Median_blur: 0.83; Majority_filter: 0.82; Connected_component_filtering: 0.82), and Case 3 (Median_blur: 0.81; Majority_filter: 0.80; Connected_component_filtering: 0.80).
